# High-resolution genetic mapping with pooled sequencing

**DOI:** 10.1186/1471-2105-13-S6-S8

**Published:** 2012-04-19

**Authors:** Matthew D Edwards, David K Gifford

**Affiliations:** 1 Computer Science and Artificial Intelligence Laboratory, Department of Electrical Engineering and Computer Science, Massachusetts Institute of Technology, Cambridge, MA 02139, USA; 2 Whitehead Institute for Biomedical Research, Cambridge, MA 02142, USA; 3 Broad Institute of MIT and Harvard, Cambridge, MA 02142, USA

## Abstract

**Background:**

Modern genetics has been transformed by high-throughput sequencing. New experimental designs in model organisms involve analyzing many individuals, pooled and sequenced in groups for increased efficiency. However, the uncertainty from pooling and the challenge of noisy sequencing data demand advanced computational methods.

**Results:**

We present MULTIPOOL, a computational method for genetic mapping in model organism crosses that are analyzed by pooled genotyping. Unlike other methods for the analysis of pooled sequence data, we simultaneously consider information from all linked chromosomal markers when estimating the location of a causal variant. Our use of informative sequencing reads is formulated as a discrete dynamic Bayesian network, which we extend with a continuous approximation that allows for rapid inference without a dependence on the pool size. MULTIPOOL generalizes to include biological replicates and case-only or case-control designs for binary and quantitative traits.

**Conclusions:**

Our increased information sharing and principled inclusion of relevant error sources improve resolution and accuracy when compared to existing methods, localizing associations to single genes in several cases. MULTIPOOL is freely available at http://cgs.csail.mit.edu/multipool/.

## Background

Advances in high-throughput DNA sequencing have created new avenues of attack for classical genetics problems. A robust method for determining the genetic elements that underlie a phenotype is to gather and group individuals of different phenotypes, interrogate the genome sequences of each group, and identify elements that are present in different proportions between the groups. We describe MULTIPOOL, a multi-locus method for analyzing high-throughput DNA sequencing reads obtained from large pools of phenotypically-extreme individuals.

### Targeted experiments

**Figure 1:**
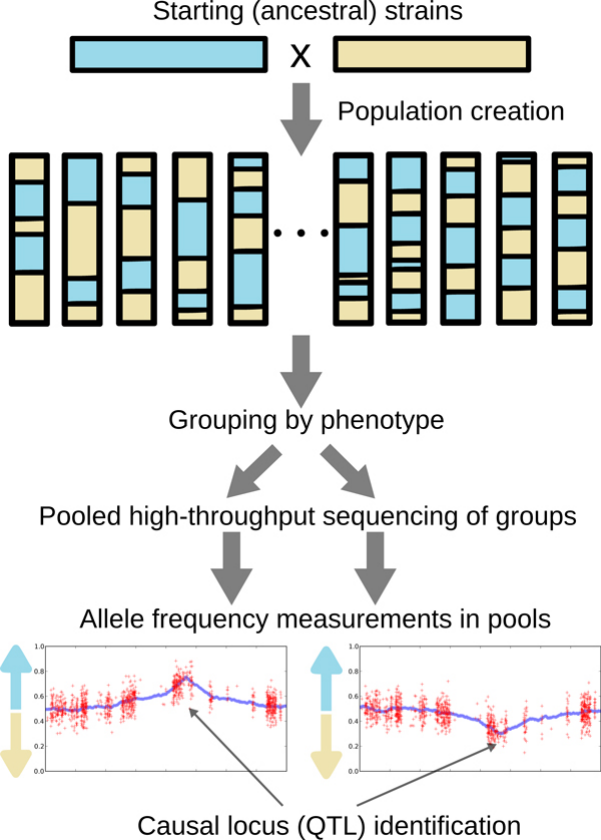
**Experimental design example**. Strains are crossed and hybrid progeny are collected. The progeny are grouped by phenotype and the pooled DNA of each group is subjected to high-throughput DNA sequencing. Loci that affect the phenotype show an enrichment for one strain in each pool, while other unlinked loci segregate evenly. The bottom two plots show simulated (unobserved) allele frequencies in the pool with blue lines and (observed) allele frequencies computed from simulated 50X sequencing coverage in red.

We focus on model organism experiments where two strains are crossed and the progeny are grouped and pooled according to phenotype. We describe and model experiments for haploid organisms that are hybrids between two strains, but we note that the models we develop should generalize to more sophisticated crosses or diploid organisms. When two strains vary in a phenotype, analyzing progeny with extreme phenotypes should elucidate the genetic basis of the trait. The main idea is that polymorphic loci that do not affect the phenotype will segregate with approximately equal frequency in the progeny (regardless of phenotype), while loci that influence the trait will be enriched in opposite directions in the extreme individuals, according to the effect size of each locus. This approach assumes that the causal loci have sufficiently strong main effects to be detectable via any type of pooled analysis. This pooled study design is also referred to as "bulk segregant analysis" [1] in model system genetics. Selection and pooling based on a quantitative phenotype can identify quantitative trait loci (QTLs), so this procedure can also be viewed as a type of pooled QTL mapping. Figure 1 illustrates the experimental design at a broad level, though there are many ways to design crosses and experimental selections to produce pools that may be analyzed by MULTIPOOL.

Bulk segregant analysis with high-throughput sequencing has been applied in yeast to study drug resistance in [2], high temperature growth in [3], and viability on alternate carbon sources in [4]. Related pooled sequencing experiments used fly [5] and *Arabidopsis *[6] model systems. In human, analogous pooled sequencing studies currently require target capture methods and a preselected set of candidate loci [7].

Pools may be selected from a single phenotypic extreme, opposite extremes, or one extreme and a control sample. Pools may also be obtained by grouping based on binary traits rather than quantitative phenotype extremes. Early studies used microarrays for pooled genotyping [8-10], but recent developments in high-throughput sequencing allow its use as a direct genotyping platform. While genotyping or sequencing individuals is an alternate choice, the appeal of pooled analysis is the dramatic reduction in cost while maintaining the statistical power of large sample sizes. See [11-13] for a discussion on pooled association studies and experiment design considerations.

### Challenges

Pooled genetic mapping studies using high-throughput sequencing present a number of unique difficulties. The core statistical quantity of interest, the allele frequency in each pool, is observed only indirectly. The strain-specific read counts that are used to estimate the allele frequencies are corrupted by sampling noise at most reasonable sequencing depths, read mapping errors [14], reference genome inaccuracies, and biological bias during sample preparation. In addition, the allele frequency measurements are nonuniformly spaced along the genome, depending on the polymorphism structure between the strains of interest. As an illustration, we refer to the bottom two plots in Figure 1 which show simulated 50X average sequencing coverage using polymorphisms from two yeast strains. Linkage implicates a wide region along the shown chromosome, and the allele frequencies estimated from read counts are noisy and not necessarily highest at the exact location of the causal allele.

However, the unbiased nature of genotyping via high-throughput sequencing results in nearly saturated marker coverage where almost all polymorphisms are queried. This avoids the laborious process of marker discovery and assay design required by earlier genotyping technologies. The dense marker coverage also allows for a high degree of information sharing, which motivates the methods underlying MULTIPOOL.

### Previous statistical methods

Previous statistical approaches to analyzing pooled genotyping data have focused on alternate regimes where genetic markers are relatively sparse and measurements are relatively accurate. Often, only single loci are tested for association, necessarily ignoring data from nearby markers. Additionally, single-locus methods encounter difficulties with missing data, such as regions that are difficult to sequence or map or have very few polymorphisms.

Earlier work applied hidden Markov models (HMMs) to fine mapping within small regions with fewer number of markers [15,16], and was extended to pooled genotype measurements in similar scenarios [17]. However, these methods relied on computationally intensive sampling methods and were applied to datasets with only a few dozen markers. Conceptually similar methods have been explored for human studies, focusing on utilizing haplotype structure in the analysis of pooled experiments [18]. In more recent pooled sequencing experiments, a sliding-window method was applied on *p*-values from local tests in [2], while a local weighted method motivated by a probabilistic model was given in [3]. However, these models do not explicitly model the location of the causal locus while considering all relevant marker data.

### Approach

MULTIPOOL is designed for experimental crosses and dense noisy genotyping, as obtained by sequencing, and handles datasets with tens or hundreds of thousands of markers. We develop a statistical model that can combine information across many nearby markers while accounting for the nonuniform noise levels introduced by varying sequencing depth and marker spacing. The specific advances we present with MULTIPOOL include:

• A model-based framework that allows for information sharing across genomic loci and incorporation of experiment-specific noise sources. These methods improve on previous approaches that rely on heuristic techniques to select sliding window sizes, which may sacrifice resolution.

• Statistical tests using an information-sharing dynamic Bayesian network (DBN) that report robust location estimates and confidence intervals. The multi-locus methods allow for principled inference even in regions without strain-specific markers and reduce experimental noise when many markers are available.

• Extensions of our method to any number of replicates and multiple experimental designs, within the same principled statistical framework.

## Methods

We develop inference methods for the pool allele frequency at a particular genome position, given the pooled read samples. First, we propose generative models which describe the experimental process. Next, these models are used to construct likelihood-based statistics to assess the significance of associations in multiple experimental designs.

### Obtaining allele frequency measurements

All sequencing reads from a particular pooling experiment are aligned to one strain's reference genome using the short read aligner bwa[19]. To increase specificity, only uniquely-mapping reads are considered. In practice, any short read aligner that can produce or export its output to the standard SAM format is compatible with this workflow. Next, a whole-genome pileup is generated using samtools[20]. A genome pileup lists the particular base calls at each genomic position, using the set of mapped sequencing reads. The genome pileup produces reference and non-reference allele counts at each base. Using single-strain sequencing data, lists of polymorphic bases can be determined and extracted from the pileup of the pooled experiments. The result is a list of allele-specific read counts at many polymorphic sites across the genome. The coverage of the marker sites will vary according to local sequencing depth and mappability [14], and the density will vary according to the local polymorphism level. A similar approach was applied to generate allele counts in [2].

### Multi-locus model

MULTIPOOL uses a probabilistic model that considers one chromosome at a time and explicitly models the effect that recombination and pool size have on neighboring allele frequencies. The model is a dynamic Bayesian network that describes the changing allele frequencies in the pool along a chromosome. The chromosome of interest is segmented into discrete blocks of equal size. A hidden state corresponding to each block reflects the pool allele frequency in the pool at that locus, varying along the genome as recombination causes random fluctuations. Each locus may emit sequencing reads according to its local pool allele frequency (hidden state). These reads may originate from multiple markers falling within the same region or a single marker. When there are no polymorphisms or mappable reads available in a region, the locus has no emissions and therefore the observed data do not directly constrain the hidden state at that locus. Finally, a particular locus may include the causal gene and therefore be directly associated with the phenotype. We assume there is only one causal locus in the analyzed region. For the genetic mapping problem, the causal locus is unknown and the key inference task is identifying its location and degree of association with the phenotype.

### Model specification

**Figure 2:**
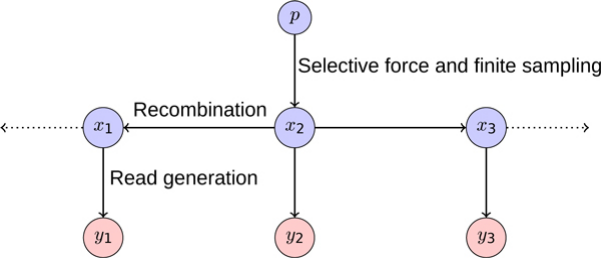
**Graphical model showing multi-locus dependencies**. Dynamic Bayesian network used by MULTIPOOL to capture the dependence between nearby loci in a pooled sequencing experiment. Allele frequencies in the pool influence the mix of observed sequencing reads at each locus. Here, the causal allele is *x*_2 _and its value is determined by sampling *N *individuals to create the pool from the population allele frequency *p *(where $p\neq \frac{1}{2}$ indicates association).

The pool is composed of *N *individuals. An unknown causal locus is linked to the phenotype and displays association with allele frequency $p\neq \frac{1}{2}$ in the population. Loci that are not associated with the phenotype and are not linked to the causal locus segregate at frequency $p=\frac{1}{2}$ in the population. The pool allele frequencies are unobserved and are given for each genome segment *i *by *x*_*i*_, *i *= {1, .., *L*}. The observed allele frequency measurements *y*_*i *_are obtained from the mapped sequencing reads. We also define *d*_*i*_, the total informative reads at each locus. This quantity is determined by the local sequencing depth and number of mappable polymorphisms. The recombination frequency *r *gives the probability of an odd number of crossovers between adjacent genome segments in one individual in the pool. We do not model crossover interference, and therefore assume that recombination events are independent along the genome. The dependencies encoded in this model can be expressed as a graphical model, shown in Figure 2. While the example figure shows a particular choice of the causal locus, the inference task consists of selecting among all possible choices (model structures) for the causal locus and the population allele frequency *p*. The population allele frequency is the allele frequency of the causal locus that would be observed in an infinitely-large pool (the population), and depends on the strength of the locus's association. Subsequent sections develop efficient methods for calculating likelihoods for all relevant model structures by reusing intermediate computations.

#### Emission probabilities

The probability of observing a set of sequencing reads conditioned on the pool fraction at the locus and a total informative read count *d*_*i *_can be calculated using the binomial distribution:

$$y_{i}\cdot d_{i}∼\text{Bin(}d_{i},x_{i}).$$

This formulation models the read count proportion exactly with a discrete model. An approximation, applicable to high read counts, can be obtained with a Gaussian distribution:

$$y_{i}\sim N\left(x_{i}\frac{x_{i}\left(1-x_{i}\right)}{d_{i}}\right).$$

Technical pooling variance that increases the local measurement noise, such as allele-specific PCR amplification bias, could be assumed to act in locus-independent manner and be modeled with increased variance in this expression.

#### Transition probabilities

In practice, the genome segments are chosen to be small enough so that *r *is effectively the probability of a single recombination event occurring. We can determine the transition probabilities from *x*_*i *_to *x*_*i*+1 _by considering the *k *individuals that switch from the first strain to the second and the *j *individuals of the reverse case. We know *k *~ Bin(*Nx*_i_, *r*) since each of the *Nx*_*i *_individuals with the first strain's ancestry at locus *i *will switch strain type when a recombination event occurs, with probability *r*. Similarly, *j *~ Bin(*N*(1 - *x*_*i*_),*r*). Thus:

$$x_{i+1}=x_{i}-\frac{k}{N}+\frac{j}{N}.$$

Employing normal approximations for the binomial distributions and dividing by *N*, we obtain an approximation for the transition probabilities:

$$\begin{array}{l} x_{i+1}∼x_{i}-N(x_{i}r,\frac{x_{i}r(1-r)}{N})+N((1-x_{i})r,\frac{\left(1-x_{i})r(1-r\right)}{N})\\ \;\;\;\;\;\;\;\;\;=N(x_{i}(1-2r)+r,\frac{(1-r)r}{N}). \end{array}$$

This formulation shows that the latent allele frequencies form a first-order autoregressive Gaussian process with mean $\frac{r}{1-\left(1-2r\right)}=\frac{1}{2}$ and variance $\frac{\left(1-r\right)r/N}{1-{\left(1-2r\right)}^{2}}=\frac{1}{4N}$, which can be verified with a single-locus analysis.

#### Initial probabilities

The causal locus node induces a particular distribution over hidden states, depending on the selected population allele frequency *p:*

$$x_{i}\cdot N∼\text{Bin(}N,p).$$

The normal approximation is:

$$x_{i}\sim N\left(p,\frac{p\left(1-p\right)}{N}\right).$$

### Inference: discrete model

Inference of the hidden state values can proceed outwards from the causal locus, using the conditional independence structure of the model. We describe the algorithms in terms of standard HMM techniques, but note that a more general treatment in terms of message passing is also possible.

The observed data likelihood $\text{Pr}\left(\vec{y}\right)$, conditioned on a particular causal allele at *x*_*c *_(model structure) and population allele frequency *p*, is obtained by conditioning on the values of the causal locus:

$$\text{Pr}\left(\vec{y}|p\right)=\overset{N}{\underset{j=0}{\sum }}\text{Pr}\left({\vec{y}}_{\left\{c+1,...,L\right\}}|x_{c}=\frac{j}{N}\right)\text{Pr}\left({\vec{y}}_{\left\{1,...,c-1\right\}}|x_{c}=\frac{j}{N}\right)\text{Pr}\left(y_{c}|x_{c}=\frac{j}{N}\right)\text{Pr}\left(x_{c}=\frac{j}{N}|p\right).$$

The first term in the sum operates on an HMM with rightwards arrows in its graph, while the second term operates on an HMM with leftwards arrows (see Figure 2). However, the latent states form a reversible Markov chain, allowing us to reverse the arrows in the left graphical model fragment. After this transformation, the likelihood computations for all choices of the causal node *x*_*c *_use the same graphical structure over the latent states $\vec{x}$ when conditioned on the causal node *x*_*c*_: two chains with all rightwards arrows, separated by the conditioned node *x*_*c*_. Using this fact, we can compute the desired likelihoods with intermediate computations from a single graphical model.

We compute the product of the first three terms in the sum, $\text{Pr}\left(\vec{y}|x_{c}\right)$, using the posterior distribution of *x*_*c *_computed using an HMM with no causal locus $\left(\text{Pr}\left(x_{c}|\vec{y}\right)\right)$. The posterior distributions are calculated using the forward-backward algorithm [21,22], using the transition and emission distributions given previously. The unconditional marginal distribution $\text{Pr}\left(x_{c}\right)$ is computed using the stationary distribution of the latent allele frequencies in the noncausal model.

$$\text{Pr}\left(\vec{y}|p\right)=\overset{N}{\underset{j=0}{\sum }}\text{Pr}\left(\vec{y}|x_{c}=\frac{j}{N}\right)\text{Pr}\left(x_{c}=\frac{j}{N}|p\right)=\text{Pr}\left(\vec{y}\right)\overset{N}{\underset{j=0}{\sum }}\frac{\text{Pr}\left(x_{c}=\frac{j}{N}|\vec{y}\right)}{\text{Pr}\left(x_{c}=\frac{j}{N}\right)}\text{Pr}\left(x_{c}=\frac{j}{N}|p\right)$$

Running the forward-backward algorithm requires considering all transitions in each chromosome block, leading to a runtime quadratic in the size of the pool: *O*(*N*^*2*^*L*). This dominates the cost for the final step of computing $\text{Pr}\left(\vec{y}|p\right)$ for all causal locus locations and a fixed *p*, which is *O*(*NL*). The quadratic dependence on the pool size renders the exact modeling of large pools prohibitive, motivating the continuous approximation in the next section.

### Inference: continuous approximation

The previous inference procedure applied to discrete hidden states where the pool composition is modeled exactly, but yielded inference algorithms that require time quadratic in the size of the pool. For large pools, we can relax this requirement and avoid the quadratic burden by modeling the allele frequency as a continuous value. The graphical model is linear-Gaussian since the transitions and observations are linear functions of the latent variables, subject to Gaussian noise. In a linear dynamical systems formulation, the model is:

$$\begin{array}{c} x_{i+1}=x_{i}-x_{i}r+\left(1-x_{i}\right)r+w=\left(1-2r\right)x_{i}+r+w,\\ y_{i}=x_{i}+v_{i}. \end{array}$$

Where:

$$\begin{array}{c} w\sim N\left(0,\frac{\left(1-r\right)r}{N}\right),\\ v_{i}\sim N\left(0,\frac{1}{4d_{i}}\right). \end{array}$$

The per-locus observation noise *v*_*i *_can be approximated with the sample variance from the observed *y*_*i*_, depending on *y*_*i *_and *d*_*i*_, or upper bounded by $\frac{1}{4d_{i}}$. The posterior probabilities over the continuous latent states can be calculated with the Kalman filtering and smoothing equations, analogous to the two recursive functions used to calculate the posterior probabilities for HMMs [21-23]. The Kalman filtering equations yield the conditional distribution of the latent state given the preceding observations with a recursive estimate:

$$\begin{array}{c} \text{Pr}\left(x_{i}|{\vec{y}}_{\left\{1,...,i\right\}}\right)=N\left(x_{i};\mu_{i},\overset{2}{\underset{i}{\sigma }}\right),\\ \mu_{i}=\left(1-2r\right)\mu_{i-1}+r+K_{i}\left(y_{i}-\left(1-2r\right)\mu_{i-1}-r\right),\\ \sigma_{i}^{2}=\left(1-K_{i}\right)P_{i-1}. \end{array}$$

Where:

$$\begin{array}{c} P_{i-1}={\left(1-2r\right)}^{2}\sigma_{i-1}^{2}+\frac{r\left(1-r\right)}{N},\\ K_{i}=\frac{P_{i}-1}{P_{i-1}+\frac{1}{4d_{i}}}. \end{array}$$

The recursions begin with the stationary distribution parameters:

$$\begin{array}{c} \mu_{0}=\frac{1}{2},\\ \sigma_{0}^{2}=\frac{1}{4N},\\ P_{0}=\frac{1}{4N}. \end{array}$$

The Kalman smoothing equations use the filtered results (forward estimates) to create estimates of the hidden state using the entire observation sequence, recursing backwards:

$$\begin{array}{c} \mathrm{Pr}(x_{i}|\vec{y})=N(x_{i};{\hat{\mu }}_{i},{\hat{\sigma }}_{i}^{2}),\\ {\hat{\mu }}_{i}=\mu_{i}+J_{i}{\hat{\mu }}_{i+1}-(1-2r)\mu_{i}-p),\\ {\hat{\sigma }}_{i}^{2}=\sigma_{i}^{2}+J_{i}^{2}({\hat{\sigma }}_{i+1}^{2}-P_{i}). \end{array}$$

Where:

$$\begin{array}{c} J_{i}=\frac{\sigma_{i}^{2}\left(1-2r\right)}{P_{i}},\\ {\hat{\mu }}_{L}=\mu_{L},\\ {\hat{\sigma }}_{L}^{2}=\sigma_{L}^{2}. \end{array}$$

As in the discrete section, the posterior distributions of the latent states under a null model can be used to compute the desired data likelihoods for all possible causal models. Required integrals are computed numerically using a fixed number of points. Specifically:

$$\text{Pr}\left(\vec{y}|p\right)=\overset{1}{\underset{0}{\int }}\text{Pr}\left(\vec{y}|x_{c}=j\right)\text{Pr}\left(x_{c}=j\right)\text{d}j=\text{Pr}\left(\vec{y}\right)\overset{1}{\underset{0}{\int }}\frac{\text{Pr}\left(x_{c}=j|\vec{y}\right)}{\text{Pr}\left(x_{c}=j\right)}\text{Pr}\left(x_{c}=j|p\right)\text{d}j.$$

Since the probability distributions during inference are represented with a constant number of parameters instead of a full vector (as in the discrete case), inference is more efficient. Specifically, computing the required quantities $\text{Pr}\left(x_{c}=j|\vec{y}\right)$ for all *c *requires *O*(*L*) time. This removes the dependence on the size of the pool that was present in the discrete method, allowing MultiPool to perform accurate inference in very large pools.

### Statistical tests

With these computations in place, we can compare all values of the causal locus and the trait association, measured by *p*. For each locus, we construct a likelihood ratio statistic comparing the hypotheses of association and no association:

$$LR\left(c\right)=\frac{{\text{max}}_{p^{\prime }}\text{Pr}\left(\vec{y}|p=p^{\prime }\right)}{\text{Pr}\left(\vec{y}|p=\frac{1}{2}\right)}=\underset{p^{\prime }}{\text{max}}\overset{N}{\underset{j=0}{\sum }}\frac{\text{Pr}\left(x_{c}=\frac{j}{N}|\vec{y}\right)}{\text{Pr}\left(x_{c}=\frac{j}{N}\right)}\text{Pr}\left(x_{c}=\frac{j}{N}|p=p^{\prime }\right).$$

The simplification occurs because the likelihood under the noncausal hypothesis at any locus is the same, namely $\text{Pr}\left(\vec{y}\right)$ from the noncausal HMM. A similar likelihood is obtained with the continuous model:

$$LR\left(c\right)=\underset{p^{\prime }}{\text{max}}\int_{0}^{1}\frac{\text{Pr}\left(x_{c}=j|\vec{y}\right)}{\text{Pr}\left(x_{c}=j\right)}\text{Pr}\left(x_{c}=j|p=p^{\prime }\right)\text{d}j.$$

We perform the maximization over *p' *numerically and calculate the likelihood ratio for all positions of the causal locus by reweighting the posterior probabilities.

#### Multiple experiments

We can analyze replicate experiments by forming a coupled dynamic Bayesian network. This analysis present two replicates, but the methods generalize to any number of coupled experiments. In this situation, the same sampling distribution is induced at the shared causal locus in two coupled chains. The joint data likelihood factors since the chains are conditionally independent given the selection node *p*:

$$LR\left(c\right)=\underset{p^{\prime }}{\text{max}}\frac{\text{Pr}\left({\vec{y}}_{1},{\vec{y}}_{2}|p=p^{\prime }\right)}{\text{Pr}\left({\vec{y}}_{i},{\vec{y}}_{2}|p=\frac{1}{2}\right)}=\frac{{\text{max}}_{p^{\prime }}\text{Pr}\left({\vec{y}}_{1}|p=p^{\prime }\right)\text{Pr}\left({\vec{y}}_{2}|p=p^{\prime }\right)}{\text{Pr}\left({\vec{y}}_{i}\right)\text{Pr}\left({\vec{y}}_{2}\right)}.$$

The maximization over *p' *must consider the product of the data likelihoods in the replicates. For designs where paired experiments are expected to show opposite effects, each experiment selects an optimal population allele frequency *p*. In this case, the null hypothesis is the coupled model where the two experiments share the same population allele frequency. The likelihood ratio is:

$$LR\left(c\right)=\frac{{\text{max}}_{p_{1,}p_{2}}\text{Pr}\left({\vec{y}}_{i}|p=p_{1}\right)\text{Pr}\left({\vec{y}}_{2}|p=p_{2}\right)}{{\text{max}}_{p_{3}}\text{Pr}\left({\vec{y}}_{1},{\vec{y}}_{2}|p=p_{3}\right)}=\frac{{\text{max}}_{p_{1}}\text{Pr}\left({\vec{y}}_{1}|p=p_{1}\right){\text{max}}_{p_{2}}\text{Pr}\left({\vec{y}}_{2}|p=p_{2}\right)}{{\text{max}}_{p_{3}}\text{Pr}\left({\vec{y}}_{1}|p=p_{3}\right)\text{Pr}\left({\vec{y}}_{2}|p=p_{3}\right)}.$$

The numerator is the product of two single-experiment maximizations, while the denominator is the coupled model likelihood that was presented for replicate analysis.

Using these results, MultiPool reports log_10 _likelihood ratios (LOD scores in the genetics community), maximum-likelihood estimates (MLE) of the causal locus location, and approximate credible intervals for the location of the causal locus. Assuming a uniform prior over causal locus locations, $\mathrm{Pr}(x_{c}|\vec{y}\propto \mathrm{Pr}(\vec{y}|x_{c})$ for a particular set of observations $\vec{y}$. In each case we fix *p *at its MLE, but could alternately integrate it out. Therefore, we can compute multi-locus statistics that include information from the entire dataset in experiments where multiple pools are available.

## Results

### Simulation results

**Figure 3:**
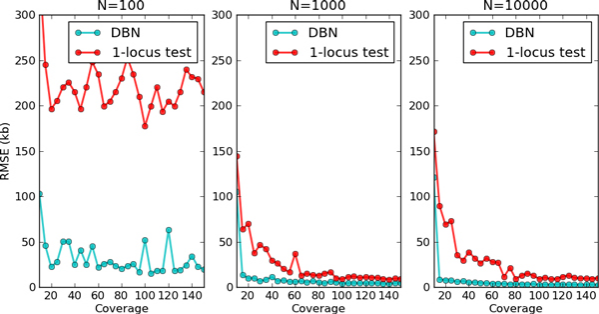
**Mapping accuracy in simulated datasets**. Mapping accuracy is shown as root mean square error (RMSE) in kilobases (kb) from the known location. The coverage reports the average sequencing depth (reads per marker) in the experiment. Each point is calculated using 100 simulated experiments. The DBN points show the accuracy of the MLE using the MULTIPOOL's one-pool test, while the 1-locus test shows the accuracy of directly testing allele frequencies calculated in 1-kb sliding windows.

In order to understand the benefit of MultiPool versus single-locus tests on deeply-sequenced pools, we conducted a series of simulations. A causal locus was chosen with population allele frequency *p = *0.75 and many pools of sizes *N = *100, 1000, and 10000 were created. SNP locations and relative per-SNP sequencing depths were calculated from experimental datasets in yeast. Average read coverage (sequencing depth) was varied from 10X to 150X, and 100 datasets of each type were simulated. The MLE causal allele location was calculated with the single-pool DBN model. The single-locus test analyzed allele frequencies computed with 1kb sliding windows, based on the method in [12]. The root mean square errors for each pool size and sequencing depth is shown in Figure 3. In the *N *= 100 cases, the mapping accuracy is predominantly controlled by the small pool size. This leads to little improvement with increased sequencing depth. The larger pools show higher accuracy with increased sequencing depth, but MultiPool is always more accurate with a lower sequencing requirement. These simulations were conducted without additional read mapping noise or other noise sources, and so the absolute results should be interpreted conservatively.

### Experimental results

We also analyze pooled sequencing data recently generated by two groups [2,3]. The groups generated haploid yeast individuals with hybrid backgrounds from two strains and performed various phenotypic selections. Table 1 lists the datasets and their sequencing depths. While each experiment generated many statistically significant novel results, we limit ourselves to mapping comparisons involving target genes that have been validated using targeted follow-up experiments. We note that even though a target gene may be verified as affecting the trait, an untested nearby gene may affect the localization results.

**Table 1:** Analyzed experiments

Name	Read length	Pool size	Coverage (rep. 1)	Coverage (rep. 2)	Source (ref.)
4-NQO viable	76	≈10000	67.7	85.0	[2]
Control	76	≈10000	36.1	79.5	[2]

Heat tolerant	76 (paired)	≈10000	152.4	84.8	[3]
Control	76 (paired)	≈10000	79.0	75.2	[3]

Each condition was assayed with two replicates. Coverage is the average reads per marker. Due to the protocols used, precise quantification of the pool size is difficult. We used the listed values as conservative choices since the reported ranges are larger in most cases.

#### Single-locus comparisons

In cases where the associated region is localized to a single gene, we compare the LOD scores from MultiPool to a likelihood ratio computed using allele frequencies calculated by summing allele read counts in sliding windows. The data likelihoods under the causal and noncausal models are calculated according to the model in [12], with the genotyping noise calculated from the local informative read depth. We use 50-bp genome segments in the dynamic Bayesian network (DBN) model and set the recombination rate in the model to the empirical average in yeast [24].

#### Large pool results

The first set of large pools was used to characterize the genetic basis of resistance to the DNA-damaging agent 4-NQO. The genes *RAD5 *and *MKT1 *were validated as affecting 4-NQO resistance with follow-up experiments, so we use them as test cases for our model. The control pools showed no association around the validated loci, so we applied MultiPool's one-pool test for association using the continuous model. Table 2 shows the distances from the MLE peak estimate to the middle of the target gene from MultiPool and sliding-window tests.

**Table 2:** Localization of known associated genes in large drug-selected pools

Dataset	Target	**DBN dist**.	**1kb window dist**.	**10kb window dist**.
4-NQO viable rep. 1	*RAD5*	5305	18355	14605
4-NQO viable rep. 2	*RAD5*	745	6195	3145

Combined	*RAD5*	805	755	3145

4-NQO viable rep. 1	*MKT1*	3223	15127	1673
4-NQO viable rep. 2	*MKT1*	5223	15127	5423

Combined	*MKT1*	4323	15127	5423

Distances are reported in bases from the MLE to the center of the target gene.

**Figure 4:**
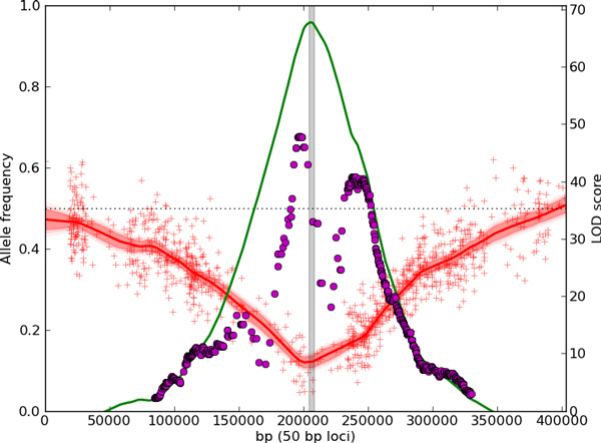
**Localization of ***RAD5 ***using 4-NQO selected replicate 2**. The red line and shaded region show the inferred allele frequencies in the pool using MULTIPOOL, and the red pluses plot the observed allele frequencies from the sequencing data (scale on left axis). Regions without pluses do not have polymorphisms or mappable reads. The magenta dots show LOD scores computed using tests of allele frequencies calculated using 10kb sliding windows, while the green line shows the LOD scores calculated using MULTIPOOL (scale on right axis). The gray box shows the position of *RAD5*, the verified causal gene in the region.

MultiPool localizes *RAD5 *to within the gene body, without a dependence on choosing an appropriate sliding window size. The 90% credible interval of the location contains six genes, centered on *RAD5*. A localization example using one replicate is shown in Figure 4. *MKT1 *is localized to within 3 kb of the gene body, with the 90% credible interval covering *MKT1 *and eight other genes.

**Figure 5:**
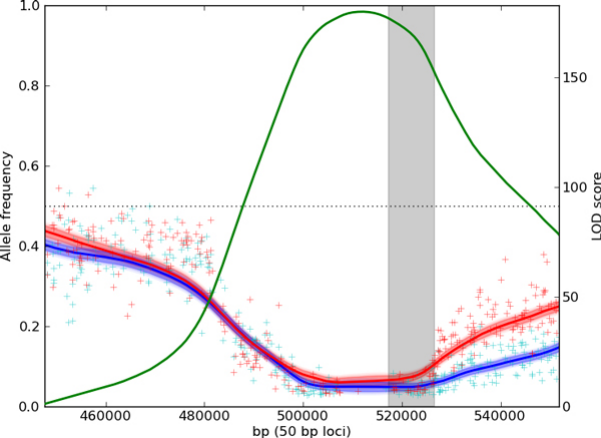
**Localization of *IRA1 *using heat tolerant replicates 1 and 2**. The red and blue lines and shaded regions show the inferred allele frequencies in the two replicates using the DBN method, and the pluses plot the observed allele frequencies. The green line shows the LOD scores calculated using the DBN two-pool method. The gray box shows the position of *IRA1*, the reported and verified association in this region. However, an uncharacterized association upstream of *IRA1 *may be the cause of the extended range of low allele frequencies and the shifted estimate of the peak location.

The second set of large pools was constructed to study the genetics of heat tolerance, using repeated crosses to reduce linkage disequilibrium (increased *r *in our model). In this study, the genes *IRA1 *and *IRA2 *were verified as affecting heat sensitivity with direct assays. Table 3 reports the distance from the MLE estimate to the center of the target gene using MultiPool and sliding window methods. *IRA2 *is localized to within the gene body, but the predictions for *IRA1 *are consistently upstream of the gene's location. Upon further investigation, the peak around *IRA1 *appears to contain another (untested) associated locus. Figure 5 plots the estimated allele frequencies and LOD scores in the surrounding region. The relevant 90% credible intervals for the causal locus location include *IRA2 *in all datasets, but do not include *IRA1*. This may support the hypothesis of another linked gene in the associated region.

**Table 3:** Localization of known associated genes in large heat-selected pools

Dataset	Target	DBN dist.	1kb window dist.	10kb window dist.
Heat tol. rep. 1A	*IRA1*	10589	16739	14739
Heat tol. rep. 1B	*IRA1*	10889	20689	6389
Heat tol. rep. 2	*IRA1*	8889	2589	17289

Heat tol. rep. 1A	*IRA2*	311	3240	511
Heat tol. rep. 1B	*IRA2*	961	17670	1661
Heat tol. rep. 2	*IRA2*	340	4190	2390

Distances are reported in bases from the MLE to the center of the target gene. The results for *IRA1 *suggest an additional associated gene; see the text and Figure 5.

## Conclusion

We presented MultiPool, a computational method to map genetic elements from pooled sequencing studies. Taking advantage of recent increases in throughput, these experimental designs use sequencing to provide unbiased and labor-efficient genotyping. As throughput continues to increase, similar studies will be extended to larger and more complex genomes. By including all relevant data in a unified framework, MultiPool improves the analysis of these experiments with increased accuracy and the principled estimation of association intervals. The statistical framework is most beneficial for the case where there are many noisy markers, as observed in genotyping via sequencing. In these cases, combining information across the genome is critical in reducing noise and increasing statistical power. More generally, the methods developed and applied in this work support the application of selection and pooled genotyping for experimental organisms. When experimental procedures can create medium or large allele frequency differences, the responsible genes can be mapped with great precision. These methods do not require the step of explicit polymorphism discovery or genotyping array design, yielding large time and cost savings.

Future work could replace our uniform prior over possible causal locus locations with an informative prior that uses conservation data, functional information, or other relevant data types (as in [25]). Other extensions include a more subtle handling of read mapping ambiguities and SNP calling uncertainty. One possibility is to use expected (average) counts under an error-aware probabilistic model instead of hard assignments, which should scale gracefully as certainty lowers. This could reduce MultiPool's reliance on a particular aligner and SNP calling strategy.

## Abbreviations

DBN: dynamic Bayesian network; HMM: hidden Markov model; LOD: base 10 logarithm of odds; MLE: maximum likelihood estimate; QTL: quantitative trait locus; RMSE: root mean square error.

## Competing interests

The authors declare that they have no competing interests.

## Authors' contributions

MDE and DKG conceived and designed the research. MDE performed the research. MDE and DKG wrote the paper.
